# The intricate role of CCL5/CCR5 axis in Alzheimer disease

**DOI:** 10.1093/jnen/nlad071

**Published:** 2023-09-28

**Authors:** Weijiang Ma, Aihua Liu, Xinya Wu, Li Gao, Jingjing Chen, Hanxin Wu, Meixiao Liu, Yuxin Fan, Li Peng, Jiaru Yang, Jing Kong, Bingxue Li, Zhenhua Ji, Yan Dong, Suyi Luo, Jieqin Song, Fukai Bao

**Affiliations:** Evidence-Based Medicine Team, Faculty of Basic Medical Sciences, The Institute for Tropical Medicine, Kunming Medical University, Kunming, Yunnan, China; Evidence-Based Medicine Team, Faculty of Basic Medical Sciences, The Institute for Tropical Medicine, Kunming Medical University, Kunming, Yunnan, China; Yunnan Province Key Laboratory of Children’s Major Diseases Research, The Affiliated Children Hospital, Kunming Medical University, Kunming, Yunnan, China; Evidence-Based Medicine Team, Faculty of Basic Medical Sciences, The Institute for Tropical Medicine, Kunming Medical University, Kunming, Yunnan, China; Evidence-Based Medicine Team, Faculty of Basic Medical Sciences, The Institute for Tropical Medicine, Kunming Medical University, Kunming, Yunnan, China; Evidence-Based Medicine Team, Faculty of Basic Medical Sciences, The Institute for Tropical Medicine, Kunming Medical University, Kunming, Yunnan, China; Evidence-Based Medicine Team, Faculty of Basic Medical Sciences, The Institute for Tropical Medicine, Kunming Medical University, Kunming, Yunnan, China; Evidence-Based Medicine Team, Faculty of Basic Medical Sciences, The Institute for Tropical Medicine, Kunming Medical University, Kunming, Yunnan, China; Evidence-Based Medicine Team, Faculty of Basic Medical Sciences, The Institute for Tropical Medicine, Kunming Medical University, Kunming, Yunnan, China; Evidence-Based Medicine Team, Faculty of Basic Medical Sciences, The Institute for Tropical Medicine, Kunming Medical University, Kunming, Yunnan, China; Evidence-Based Medicine Team, Faculty of Basic Medical Sciences, The Institute for Tropical Medicine, Kunming Medical University, Kunming, Yunnan, China; Evidence-Based Medicine Team, Faculty of Basic Medical Sciences, The Institute for Tropical Medicine, Kunming Medical University, Kunming, Yunnan, China; Evidence-Based Medicine Team, Faculty of Basic Medical Sciences, The Institute for Tropical Medicine, Kunming Medical University, Kunming, Yunnan, China; Evidence-Based Medicine Team, Faculty of Basic Medical Sciences, The Institute for Tropical Medicine, Kunming Medical University, Kunming, Yunnan, China; Evidence-Based Medicine Team, Faculty of Basic Medical Sciences, The Institute for Tropical Medicine, Kunming Medical University, Kunming, Yunnan, China; Evidence-Based Medicine Team, Faculty of Basic Medical Sciences, The Institute for Tropical Medicine, Kunming Medical University, Kunming, Yunnan, China; Evidence-Based Medicine Team, Faculty of Basic Medical Sciences, The Institute for Tropical Medicine, Kunming Medical University, Kunming, Yunnan, China; Evidence-Based Medicine Team, Faculty of Basic Medical Sciences, The Institute for Tropical Medicine, Kunming Medical University, Kunming, Yunnan, China; Yunnan Province Key Laboratory of Children’s Major Diseases Research, The Affiliated Children Hospital, Kunming Medical University, Kunming, Yunnan, China

**Keywords:** Alzheimer disease, Axis, CCL5, CCR5, Neuroinflammation

## Abstract

The morbidity and mortality associated with Alzheimer disease (AD), one of the most common neurodegenerative diseases, are increasing each year. Although both amyloid β and tau proteins are known to be involved in AD pathology, their detailed functions in the pathogenesis of the disease are not fully understood. There is increasing evidence that neuroinflammation contributes to the development and progression of AD, with astrocytes, microglia, and the cytokines and chemokines they secrete acting coordinately in these processes. Signaling involving chemokine (C-C motif) ligand 5 (CCL5) and its main receptor C-C chemokine receptor 5 (CCR5) plays an important role in normal physiologic processes as well as pathologic conditions such as neurodegeneration. In recent years, many studies have shown that the CCL5/CCR5 axis plays a major effect in the pathogenesis of AD, but there are also a few studies that contradict this. In short, the role of CCL5/CCR5 axis in the pathogenesis of AD is still intricate. This review summarizes the structure, distribution, physiologic functions of the CCL5/CCR5 axis, and the progress in understanding its involvement in the pathogenesis of AD.

## INTRODUCTION

Alzheimer disease (AD) is a chronic neurodegenerative disease that is the most common cause of dementia. It is characterized by progressive cognitive dysfunction and behavioral disorder; early symptoms such as loss of recent memories and mild cognitive decline are relatively insidious. Agnosia, aphasia, apraxia, memory impairment, and personality and behavioral changes can emerge with disease progression, which may eventually result in death. A cross-sectional survey conducted in China in 2020 reported 15.07 million cases of dementia in the population aged over 60 years, with about two-thirds (9.83 million) being AD (1). It is estimated that the number of cases of dementia will reach 130 million by 2050. Given that it accounts for over 50% of all cases of dementia (2, 3), AD will constitute a substantial healthcare burden in the future.

The pathogenesis of AD is not fully understood, and there are no specific curative treatments; existing drugs only alleviate symptoms and delay disease progression. Clarifying the pathogenesis of AD is important for the identification of novel therapeutic targets. The major histopathologic hallmarks of AD are extracellular senile plaques formed by the deposition of amyloid β (Aβ) protein and intracellular neurofibrillary tangles (NFTs) composed of tau proteins misfolded as a result of hyperphosphorylation in neurons (4). The Aβ cascade and tau protein hyperphosphorylation hypotheses have been proposed to explain AD pathogenesis. Aβ is a 38–43 amino acid polypeptide that is mainly hydrolyzed from amyloid precursor protein (APP). Under physiologic conditions, Aβ production and clearance are in dynamic equilibrium; however, under pathologic conditions, there is increased production or reduced clearance of Aβ, leading to its accumulation. Aβ monomers assemble into oligomers and polymers, mainly Aβ_40_ and Aβ_42_, and ultimately form insoluble neurotoxic amyloid plaques (5, 6). Tau is important for the assembly of the microtubule network, stabilization of microtubule structure, and regulation of axon transport in neurons. Tau exists in phosphorylated and dephosphorylated states that are normally in dynamic equilibrium. Under pathologic conditions, hyperphosphorylated tau does not bind but instead dissociates from microtubules and forms NFTs (7). However, it is unclear how abnormalities in Aβ and tau contribute to the pathogenesis of AD. For example, although drugs that clear Aβ reduce Aβ load in the brain of patients with AD, this does not translate into improvements in cognitive function (8). Various drugs have been developed for AD that prevent tau protein aggregation and stabilize microtubule structure, but clinical trials were terminated due to insufficient curative effect or toxicity. Although tau protein immunotherapy has shown therapeutic promise, its efficacy has yet to be demonstrated in clinical trials (9).

Neuroinflammation refers to an inflammatory response to trauma, infection, ischemia, toxins, etc. mediated by glial cells, vascular endothelial cells, and peripheral immune cells in the central nervous system (CNS). It involves cytokines such as interleukin-1β (IL-1β), interleukin-6 (IL-6), and tumor necrosis factor (TNF), chemokines such as C-C motif chemokine ligand 1 and C-C motif chemokine ligand 5 (CCL5), complement factors, nitric oxide (NO), and reactive oxygen species (ROS) (10). Acute neuroinflammation is a protective mechanism that helps to eliminate pathogens, resist adverse stimuli, and promote nerve cell repair. However, excessive and chronic neuroinflammation leads to the continuous release of proinflammatory cytokines and oxidative stress, which can cause tissue damage and adversely affect neuron structure and function. Neuroinflammation plays an important role in AD pathogenesis and progression (11). Chemokines are small proteins (70–90 amino acids) with a molecular weight of 8–12 kDa and varied functions, including chemotaxis of leukocytes to sites of inflammation, anti-infective activity, and regulation of the immune response. According to the number and spacing of N-terminal cysteines, chemokines can be divided into 4 subfamilies, namely, CXC, CC, C, and CX3C (12). The CCL5/C-C chemokine receptor 5 (CCR5) axis comprising CCL5 and its main receptor CCR5 is involved in multiple pathologic states such as tumors, infectious diseases, and nervous system diseases. Since CCR5 was found to be a co-receptor of HIV-infected target cells, extensive research has been carried out in HIV infection, and several CCR5 antagonists have been designed to block HIV from entering host cells (13). Although accumulating evidence indicates that CCL5/CCR5 axis can facilitate the development of AD, some studies have come to the opposite conclusion. At this time, the relationships between this pathway and AD are still unclear. This article analyzes associations between the CCL5/CCR5 axis, neuroinflammation, and AD.

## OVERVIEW OF CCL5/CCR5 AXIS

### CCL5 and CCR5 structure, distribution, and signal pathway

CCL5 (also known as regulated on activation, normal T-cell expressed, and secreted [RANTES]) is a member of the CC chemokine subfamily. The gene encoding CCL5 is located on human chromosome 17q11.2-q12. CCL5 is mainly secreted by T cells and is also expressed in platelets, macrophages, synovial fibroblasts, and hepatocytes. Other CCL5 family members include CCL3 (also known as macrophage inflammatory protein 1-α [MIP-1α]) and CCL4 (MIP-1β). CCL5 can bind to CCR1, CCR3, and CCR4 but the main receptor is CCR5 (14), a 7-transmembrane G protein-coupled receptor composed of 352 amino acids, with 3 extracellular loops and 3 intracellular loops and a molecular weight of 40.6 kDa (15). There are 4 cysteine residues in the extracellular domain that form disulfide bonds and stabilize the extracellular structure; the second intracellular loop contains a conserved sequence (DRYLAVHA) that interacts with G protein (16). CCR5 is expressed by a variety of immune cells including T cells, macrophages, and dendritic cells; in the CNS, it is expressed at a high level in microglia but at a relatively low level in neurons and astrocytes (17). Since its identification as a co-receptor that facilitates HIV entry into target cells (18, 19), CCR5 has been widely studied in the context of inflammation, tumors, infection, and diseases of the CNS and other systems.

**Figure 1.**
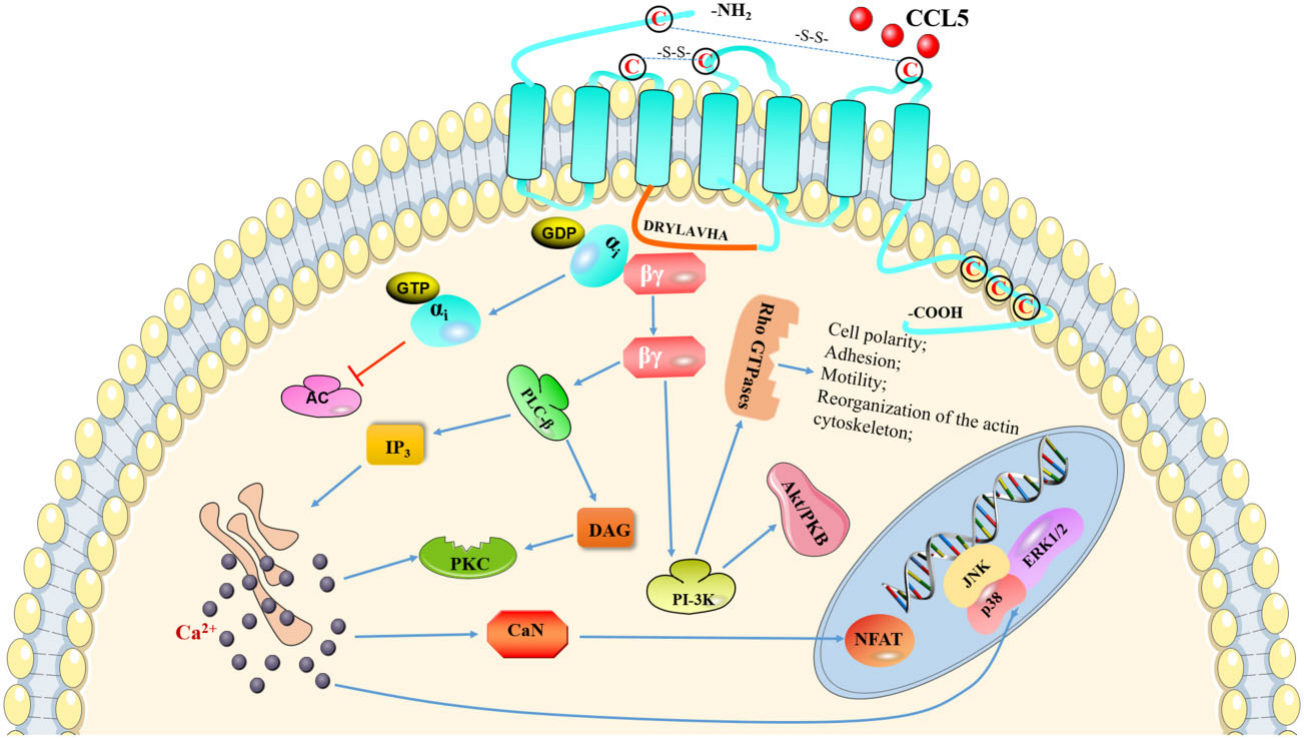
CCL5/CCR5 signaling pathway. CCR5 is a 7-transmembrane G protein-coupled receptor with 3 extracellular loops and 3 intracellular loops. The disulfide bonds formed by 4 cysteine residues in the extracellular domain are vital for stabilizing the extracellular structure. Three cysteine residues on the carboxyl terminal domain can anchor the tail to the membrane. DRYLAVHA sequence on the second intracellular loop can interact with G protein. Following CCL5 binding to CCR5, G protein dissociates into the α_i_ and βγ subunits; the former inhibits adenylate cyclase (AC), whereas the latter activates phospholipase Cβ (PLCβ) and phosphatidylinositol 3 kinase (PI-3K). The following mediators are then activated in turn: diacylglycol (DAG), protein kinase C (PKC), inositol-1,4,5-triphosphate (IP3), intracellular Ca^2+^, calcineurin (CaN), nuclear factor of activated T cells (NFAT), extracellular signal regulated kinase 1 and 2 (ERK1/2), p38, c-Jun N-terminal kinase (JNK), protein kinase B (PKB/Akt), and Rho GTPases, which eventually affect cell migration, survival, cytoskeleton rearrangement, cell polarization, and T-cell proliferation.

Following CCL5 binding to CCR5, G protein dissociates into the α_i_ and βγ subunits; the former inhibits adenylate cyclase, whereas the latter activates phospholipase Cβ (PLCβ) and phosphatidylinositol 3 kinase (20). Activated PLCβ mediates the production of diacylglycerol (DAG) and inositol-1,4,5-triphosphate (IP3); IP3 then induces the release of intracellular Ca^2+^, leading to activation of calcineurin, dephosphorylation of nuclear factor of activated T cells, and transcription and activation of mitogen-activated protein kinases (MAPKs) such as extracellular signal regulated kinase 1 and 2, p38, and c-Jun N-terminal kinase (JNK) to regulate cell migration and T-cell proliferation. Additionally, Ca^2+^ and DAG activate protein kinase C, which in turn promotes receptor phosphorylation (21, 22). Activated PI3K can also induce the activation of protein kinase B (PKB/Akt) and Rho GTPases; PI3K/Akt signaling is related to cell survival, whereas Rho GTPases participate in cytoskeleton rearrangement and regulate cell polarization, adhesion, and movement (16, 23, 24). The CCL5/CCR5 signaling pathway is summarized in Figure 1.

### Physiologic functions of the CCL5/CCR5 axis in the brain

In addition to the immune response, CCR5 is involved in neurotransmission, brain development, and learning and memory, among other functions (25). CCR5 is expressed throughout the CNS at all stages of development from embryo to adult, but the distribution varies across brain areas, which may be related to its different functions in different parts of the brain (26, 27). CCR5 is expressed in glial cells and neurons in the cerebral cortex, hippocampus, basal nuclei, and thalamus, and the expression level was shown to increase from birth to 9 months of age, and it was also observed in adult neural progenitor cells (28), which indicates that CCR5 is involved in neural development. Additionally, in the aspect of neurotransmission, CCL5 modulates the release of glutamate from neurons via CCR5 (29). Learning and memory have always been an attractive research direction in the field of neuroscience. CCR5 has had a great impact on learning and memory, resulting in many related studies. For example, a study has shown that CCR5 may affect normal learning and memory by acting on the cyclic AMP response element-binding (CREB) protein pathway (30). CCR5 was also shown to reduce the plasticity of neurons in the cerebral cortex and hippocampus-dependent learning and memory. Therefore, the effect of activating or inhibiting CCL5/CCR5 axis on memory may be opposite, and some studies have also proved this. Inhibition of CCR5 increased MAPK/CREB signaling and enhanced memory, whereas CCR5 overexpression resulted in memory impairment and decreased plasticity (31). It was also reported that CCL5 and CCR5 overexpression during aging was associated with memory impairment in mice, which was reversed by knocking out the *CCR5* gene through gene editing or pharmacologic inhibition with maraviroc (32). However, activation of CCR5 by CCL3 impaired synaptic plasticity of hippocampal neurons and memory, which can be alleviated by application of the CCR5 inhibitor maraviroc (33). Thus, the above evidence indicates that CCL5/CCR5 axis plays an important role in neurocognitive function, which also provides potential clues for exploring the pathogenesis of disorders associated with cognitive deficits.

## CCL5/CCR5 AXIS IN AD

The neuroinflammation hypothesis of AD posits that microglia and astrocytes are activated by Aβ deposition and undergo phenotypic and functional transformation to remove Aβ and cell fragments; however, the secretion of cytokines, chemokines, ROS, and NO during Aβ removal induces oxidative stress and inflammation, which can cause neuronal damage and promote AD progression (10, 34). For a long time in the past, reactive astrocytes and microglia were thought to have 2 phenotypes and play different functions under different pathological conditions. For reactive astrocytes, the toxic A1 type releases proinflammatory cytokines and promotes neuronal damage, and the A2 type protects neurons by secreting anti-inflammatory cytokines and neurotrophic factors (35). Microglia have processes that sense harmful signals in the environment (36). Aβ activation of microglia results in changes in microglia morphology and function (37). M1 microglia secrete IL-1β, TNF-α, interferon-γ (IFN-γ), and CCL2; activate inducible NO synthase (iNOS); and induce the production of ROS to promote neuroinflammation. Meanwhile, M2 microglia release interleukin (IL)-4, IL-10, IL-13, and neurotrophic factors that have anti-inflammatory and antioxidant functions and promote the repair of cell and tissue injury to restore brain homeostasis (38, 39). However, we should emphasize that this superficial dichotomy theory has many limitations, which greatly simplifies the functional diversity of neuroglia. With the popularization of whole-genome transcriptomics, epigenomics, and proteomics in the research of reactive astrocytes and microglia, many new evidences show that their different subtypes may coexist in the CNS, and their biological functions are diverse and rich, involving novel regeneration, neural stem cell potential, regulating overactive neurons, synaptic pruning, promoting neuronal survival, and many other aspects (40, 41).

CCR5 is significantly upregulated in reactive microglia in patients with AD, which was shown to be associated with Aβ deposition (42). One study found that RANTES level was significantly higher in patients with AD than in control subjects and was positively correlated with IL-6 and TNF-α levels (43), implying that CCL5 contributes to AD pathogenesis by mediating the inflammatory response and is a potential biomarker for early disease diagnosis. Genome-wide analyses have also identified CCR5 as one of the major hub genes in AD (44). In a rat model of lipopolysaccharide (LPS)-induced neuroinflammation, the CCR5 antagonist d-Ala-peptide T-amide reduced the number of microglia and astrocytes in the hippocampus as well as astrocyte hypertrophy, and microglia had slender processes similar to those observed in the nonactivated state (45). In CCR5^−/−^ mice, Aβ_1–40_ overexpression in hippocampus decreased the aggregation of astrocytes and microglia and alleviated cognitive impairment and synaptic dysfunction; these effects were associated with the downregulation of cyclooxygenase-2, iNOS, and nuclear factor κB (46). These findings suggest that blocking CCR5 can reduce neuroinflammation, which may be an effective therapeutic strategy for AD. As for the mechanism of CCR5 overexpression in AD, it was found that Aβ promoted CCR5 expression by enhancing the binding of transcription factor early growth response protein 1 (Egr-1) to the *CCR5* promoter; *Egr-1* gene silencing inhibited CCR5 expression and abolished chemotaxis mediated by CCR5 (47). Thus, Aβ may induce CCR5 expression in microglia. As immune cells of the CNS, activated microglia release many soluble factors such as TNF-α, NO, IL-10, and insulin-like growth factor-1 (IGF-1). An in vitro experiment showed that CCL5 increased NO secretion by activated microglia and reduced IL-10 and IGF-1 production, indicating that CCL5 not only exerts a chemotactic effect but also regulates microglia to enhance neuroinflammation (48).

**Figure 2.**
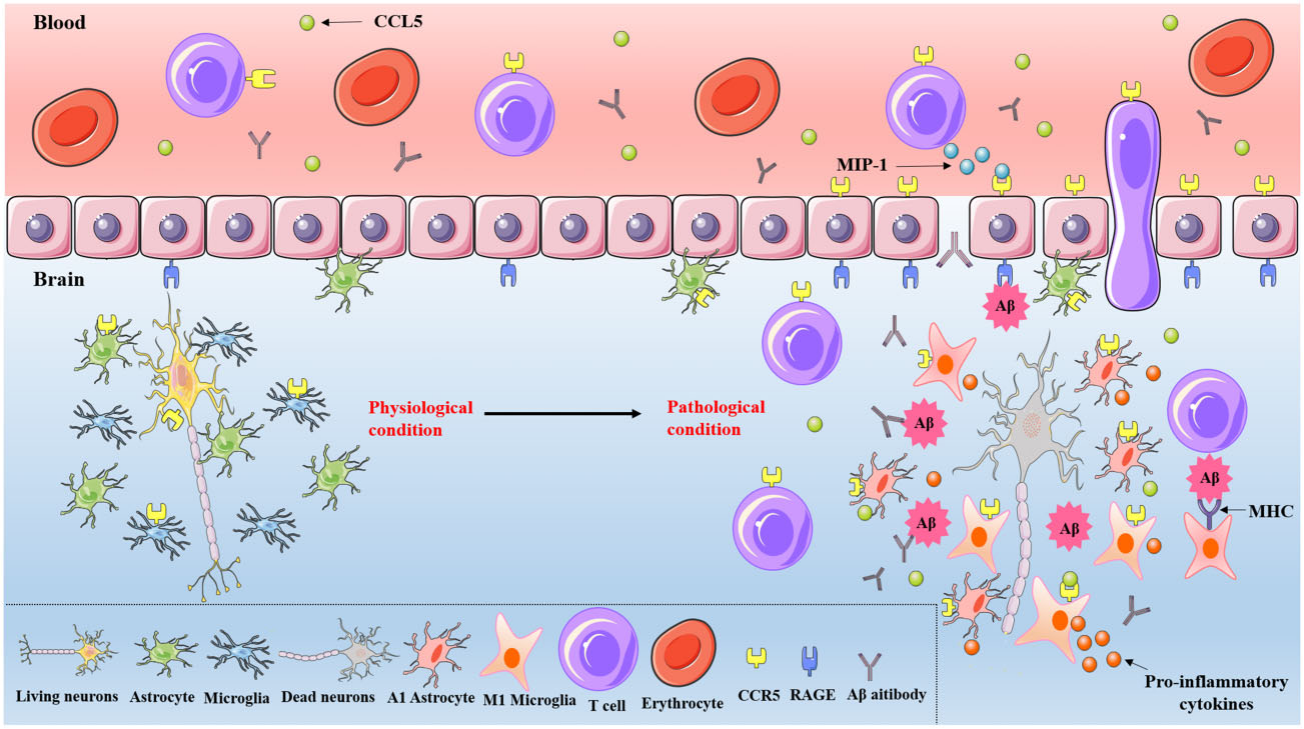
Mechanisms of the CCL5/CCR5 axis in the pathogenesis of AD. In patients with AD, Aβ can upregulate CCL5 and CCR5; the resting microglia and astrocytes are transformed into the A1 and M1 phenotypes, and they secrete pro-inflammatory cytokines. Aβ deposition in the brain leads to immune activation and production of antibodies. Interaction of Aβ and receptor for advanced glycation endproducts (RAGE) expressed by cells of the blood-brain barrier (BBB) can promote *CCR5* expression, resulting in the penetration of MIP-1-expressing T cells through tight junctions of the BBB. There is crosstalk between T cells and microglia, microglia act as antigen-presenting cells to present Aβ to T cells, which in turn induce microglia differentiation. With the development of AD, neurons eventually die.

The CCL5/CCR5 axis also mobilizes peripheral immune cells to participate in the immune response in AD. Aβ deposition in the brain leads to immune activation and production of antibodies that promote the clearance of Aβ (49). Besides antibody-mediated humoral immunity, T-cell-mediated cellular immunity is also involved in AD pathogenesis. T cells in patients with AD are activated and exist both in the periphery and infiltrate the brain (50). Additionally, a flow cytometry study analyzing the phenotypic profile of circulating immune cells in AD found that the proportion of immune cells expressing CCR5 was higher in AD patients than in age-matched controls and that CCR5 was more highly expressed on CD4^+^ versus CD8^+^ T cells (51). CCR5 expression was almost undetectable in peripheral blood mononuclear cells (PBMCs) of healthy subjects but was upregulated in patients with AD (52). The amount of CCL5 released by PBMCs was also higher in the AD group than in the control group. Treatment with the acetylcholinesterase inhibitor pyridostigmine decreased CCL5 and CCR5 expression in PBMCs of AD patients; this was reversed by overexpression of Aβ_1–42_, which also increased IFN-γ expression but decreased that of IL-4. There is also evidence of crosstalk between T cells and microglia. Microglia act as antigen-presenting cells to present Aβ to T cells, which in turn induce microglial differentiation (53, 54). However, it is unclear how T cells cross the blood-brain barrier (BBB) to enter the brain in individuals with AD. One study found that T cells in patients with AD overexpress MIP-1, which binds to CCR5 expressed by brain endothelial cells to facilitate the passage of T cells through tight junctions of the BBB (55). Furthermore, interaction of Aβ and receptor for advanced glycation endproducts (RAGE) expressed by cells of the BBB can activate ERK, JNK, and PI3K signaling to enhance the binding of Egr-1 to the *CCR5* promoter and expression of *CCR5*, resulting in the penetration of MIP-1-expressing T cells through the BBB (56). Thus, Aβ deposition acts as a signal that is transmitted via RAGE to peripheral T cells, which then promote AD development. These results indicate that the interaction between Aβ and the CCL5/CCR5 axis not only perturbs immune homeostasis in the CNS but also modulates systemic immunity to promote pathologic processes in AD (summarized in Fig. 2).

There have been a few studies in both animal models and in human subjects that do not support the view that CCL5/CCR5 axis can promote the development of AD. CCR5 was shown to be downregulated in the APP/prenilin-1 transgenic mouse model of AD (57); compared with control subjects, *CCL5* mRNA level in the peripheral blood was significantly lower in AD patients (58). Meanwhile, there was no difference in *CCL5* expression between AD model and wild-type mice (59). These results argue against a significant role for the CCL5/CCR5 axis in AD development. However, the contribution of the CCL5/CCR5 axis to AD pathogenesis cannot be extrapolated based solely on alterations in the expression of pathway components. There is also evidence that the CCL5/CCR5 axis has a protective role in AD. One study using CCR5^−/−^ mice showed that *CCR5* deficiency accelerated LPS-induced astrogliosis activation and Aβ deposition and impaired memory function (60). Similarly, another study found that astrocyte activation, Aβ_1–42_ deposition, and memory impairment were enhanced in *CCR5*-deficient mice compared with wild-type mice (61). In vitro experiments have shown that RANTES application improved the survival rate of neurons and increased their tolerance to the toxicity of sodium nitroprusside and thrombin (62), suggesting that RANTES exerts a neuroprotective effect. Additionally, transplantation of bone marrow mesenchymal stem cells into the brain of AD mice improved cognitive function, which was attributed to the action of secreted CCL5 on endogenous microglia (63).

The relationship between CCR5 and AD has been investigated from the perspective of genetic diversity, but most studies have found that the distribution of *CCR5Δ32* allele does not differ between AD patients and healthy individuals (64–67). However, a large-scale cohort study reported that although there was no significant association between *CCR5Δ32* polymorphism and the screened neurodegenerative diseases, carriers of the *CCR5Δ32* allele had an earlier disease onset (68).

## CONCLUSION

There is ample evidence that the immune response is involved in the pathogenesis of AD; the neuroinflammation hypothesis is highly attractive and is a powerful complement to the Aβ and tau protein hypotheses. During neuroinflammation, Aβ deposition activates astrocytes and microglia, with the latter transforming from a protective to a proinflammatory phenotype. Acute inflammation at the early stage is beneficial for clearing Aβ and repairing neuronal damage. However, with the progression of AD, microglia and astrocytes secrete proinflammatory cytokines and chemokines, leading to excessive and uncontrolled inflammation and neuron death. The CCL5/CCR5 axis plays an important role in learning, memory, neuroinflammation, and AD pathogenesis; however, the mechanistic details of CCL5/CCR5 axis in AD have not been fully elucidated and some of the existing evidence is contradictory. Even population-based studies on the distribution of the *CCR5Δ32* allele have shown no association between CCL5/CCR5 and AD development. A reason for these conflicting findings may be differences in animal models and populations, but it is also possible that CCR5 has as-yet unidentified functions in the CNS. More studies are needed to explore the specific role of the CCL5/CCR5 signaling axis in AD pathogenesis in order to determine whether it can serve as a therapeutic target in treatment of AD. In addition, we believe that it will also be a potentially valuable idea to expand the scope of exploring CCL5/CCR5 axis to other neurodegenerative diseases.
